# A three-step approach identifies novel shear stress-sensitive endothelial microRNAs involved in vasculoprotective effects of high-intensity interval training (HIIT)

**Published:** 2019-06-04

**Authors:** Boris Schmitz, Franziska L. Breulmann, Bothaynah Jubran, Florian Rolfes, Lothar Thorwesten, Michael Krüger, Andreas Klose, Hans-Joachim Schnittler, Stefan-Martin Brand

**Affiliations:** ^1^ Institute of Sports Medicine, Molecular Genetics of Cardiovascular Disease, University Hospital Muenster, Muenster, Germany; ^2^ Institute of Anatomy and Vascular Biology, University of Muenster, Muenster, Germany; ^3^ Department of Physical Education and Sports History, University of Muenster, Muenster, Germany

**Keywords:** miRNA, shear stress, performance, endothelium, high-intensity training (HIT)

## Abstract

Circulatory microRNAs (c-miRNAs) are regulated in response to physical activity and may exert anti-atherosclerotic effects. Since the vascular endothelium is an abundant source of c-miRNAs, we aimed to identify novel vasculoprotective exercise-induced c-miRNAs by the combined analysis of published endothelial miRNA array data followed by *in vivo* and *in vitro* validation. We identified 8 different array-based publications reporting 185 endothelial shear stress-regulated miRNAs of which 13 were identified in ≥3 independent reports. Nine miRNAs had already been associated with physical activity. Of the remaining novel miRNAs, miR-98-3p and miR-125-5p were selected for further analysis due to reported vasculoprotective effects. Analysis in two different 4-week high-intensity interval training (HIIT) groups (group 1 [n=27]: 4x30 s, group 2 [n=25]: 8x15 s; all-out running) suggested significantly elevated miR-98 and miR-125a-5p levels in response to acute exercise at baseline and at follow-up. Endothelial *in vitro* shear stress experiments revealed increased miR-125a-5p and miR-98-3p levels in medium of human umbilical vein endothelial cells at 30 dyn/cm^2^ after 20 and 60 min, respectively. Our results suggest that miR-98-3p and miR-125a-5p can be rapidly secreted by endothelial cells, which might be the source of increased c-miR-98-3p and -125a-5p levels in response to HIIT. Both miRNAs attenuate endothelial inflammation and may mediate vasculoprotective effects of physical exercise including HIIT.

## INTRODUCTION

microRNAs (miRNAs) have been identified as pivotal modulators of the systemic response to physical exercise and subsequent training adaptations [1-4]. Since the general knowledge on miRNAs and their specific targets and functions has greatly increased (see [5] for comprehensive review), miRNAs may also hold the potential to serve as functional biomarkers indicating physiological processes involved in the response to specific training regimes [1, 2, 4]. miRNAs are short (~21 - 23 nucleotide-long) non-coding RNAs involved in translational repression [6, 7] regulating a wide range of different physiological processes including development and aging as well as disease [8-10]. Moreover, it has been estimated that up to 60 % of all human protein-coding genes are conserved miRNA targets [11]. The myocardium and vascular endothelium are an abundant source for miRNAs that are selectively secreted into the blood stream where they can be detected as circulating miRNAs (c-miRNAs) [12]. These c-miRNAs are preserved by association with RNA-binding proteins or small membranous vesicles and commonly involved in inter-cell communication with active regulation of target cell gene expression [13, 14]. c-miRNA production and secretion is responsive to different stimuli induced by physical exercise including shear stress and hypoxia [15-17]. To this respect, it has been suggested that induction of hemodynamic stimuli including transmural pressure and (episodic) shear stress [18-20] may be key mechanisms responsible for the beneficial impact of physical exercise on vascular function [21, 22]. It has also been noted that the vascular endothelium is an important ‘mechano-sensor’ transducing hemodynamic signals which may result in flow-induced conversion of endothelial cells into an elongated arterial phenotype as well as in functional and structural changes of the overall arterial wall [20, 23]. Of note, vascular maladaptations including endothelial cell stiffening, disturbed endothelial barrier function and reduced nitric oxide (NO) production [24, 25] as well as the development of atherosclerotic lesions and plaque formation is mainly found in regions with disturbed flow which increases the secretion of pro-inflammatory molecules and most likely alters miRNA expression [26, 27]. By contrast, these deleterious changes are mostly absent from regions with constant laminar flow [26]. While *in vitro* and *ex vivo* shear stress experiments have linked an increase in mean shear stress (i.e. constant laminar shear stress) to local anti-atherosclerotic changes, it has been noted that beneficial effects of exercise on vascular function also occur in arteries that are not subjected to a direct increase in shear stress [18, 23]. To this end, selectively released miRNAs preserved by association with small membranous vesicles or RNA-binding proteins may be involved [13, 14]. These distal effects might include, for example, miRNA-dependent regulation of endothelial proliferation as shown for miR-126 [28], vascular smooth muscle plasticity as reported for miR-145/ -143 [29] and many more [15]. However, the process and molecular mechanisms involved in miRNA expression regulation and secretion, especially in response to physical exercise, is still incompletely understood [30, 31]. It has been suggested that miRNA-releasing cells may possess a sorting mechanism guiding specific miRNAs to enter exosomes resulting in the concentration of selected miRNAs [32]. To this end, *in vitro* shear stress experiments have been shown to alter not only the quantity of exosomes but also the protein and/ or (mi)RNA content of exosomes derived from endothelial cells [33, 34].

We have recently used different high-intensity interval training (HIIT) protocols to characterize conditions which lead to the expression of established c-miRNAs such as miR-126, -222 and -29c [35, 36]. Compared to endurance training, HIIT is marked by brief bursts of near-maximal to supra-maximal work rates followed by short periods of rest or active recovery accompanied by an overall reduction in training duration [37, 38]. Since the optimal HIIT conditions in terms of intensity and work/rest ratio are still under debate [38-40], miRNAs may also be used to indicate most effective HIIT variants. This might also be of interest since HIIT has been shown as efficient tool to improve health-related fitness in the general population [37, 39, 41] and for the prevention of lifestyle-induced chronic diseases [39, 42, 43].

**Figure 1:**
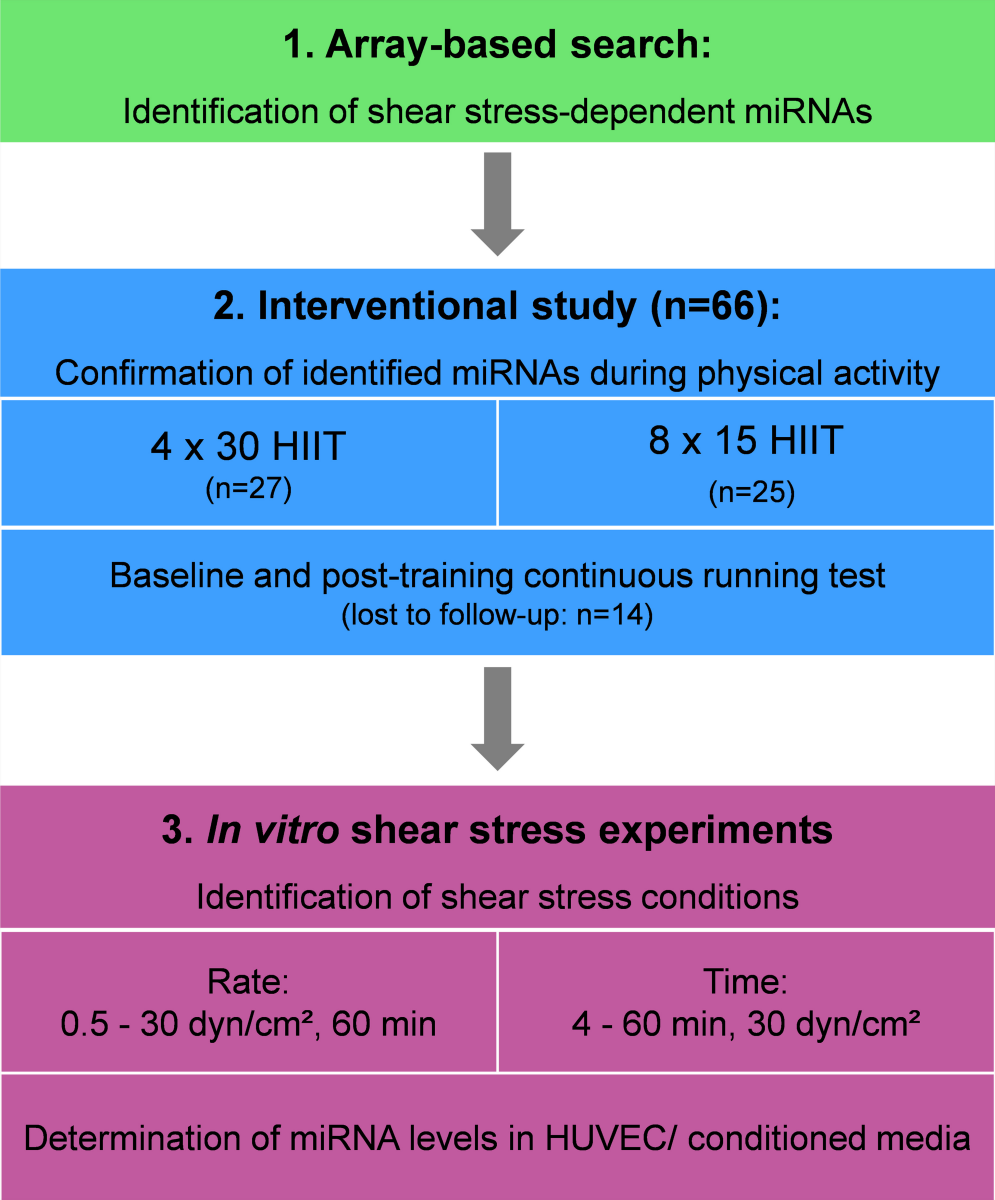
Overall study design. HIIT, high-intensity interval training; HUVEC, human umbilical vein endothelial cells.

While recent progress in array and sequencing technologies has entered the field of exercise physiology to identify novel exercise-dependent miRNAs, already available data sets might be used for the discovery of new exercise-inducible miRNAs. We hypothesized that a combined analysis of published miRNA array results from endothelial shear stress experiments would result in the identification of so far unreported miRNAs that are inducible by high-intensity exercise and may be involved in vasculoprotective effects of HIIT. Thus, the current investigation was based on a three-step study design (Figure 1) including identification of published miRNA array data sets from endothelial shear stress experiments, analysis of identified candidate miRNAs in two different HIIT groups of healthy individuals and validation of identified miRNAs at different shear rates and time points using an *in vitro* endothelial shear stress model.

## RESULTS

### Search results and identified miRNAs

**Figure 2:**
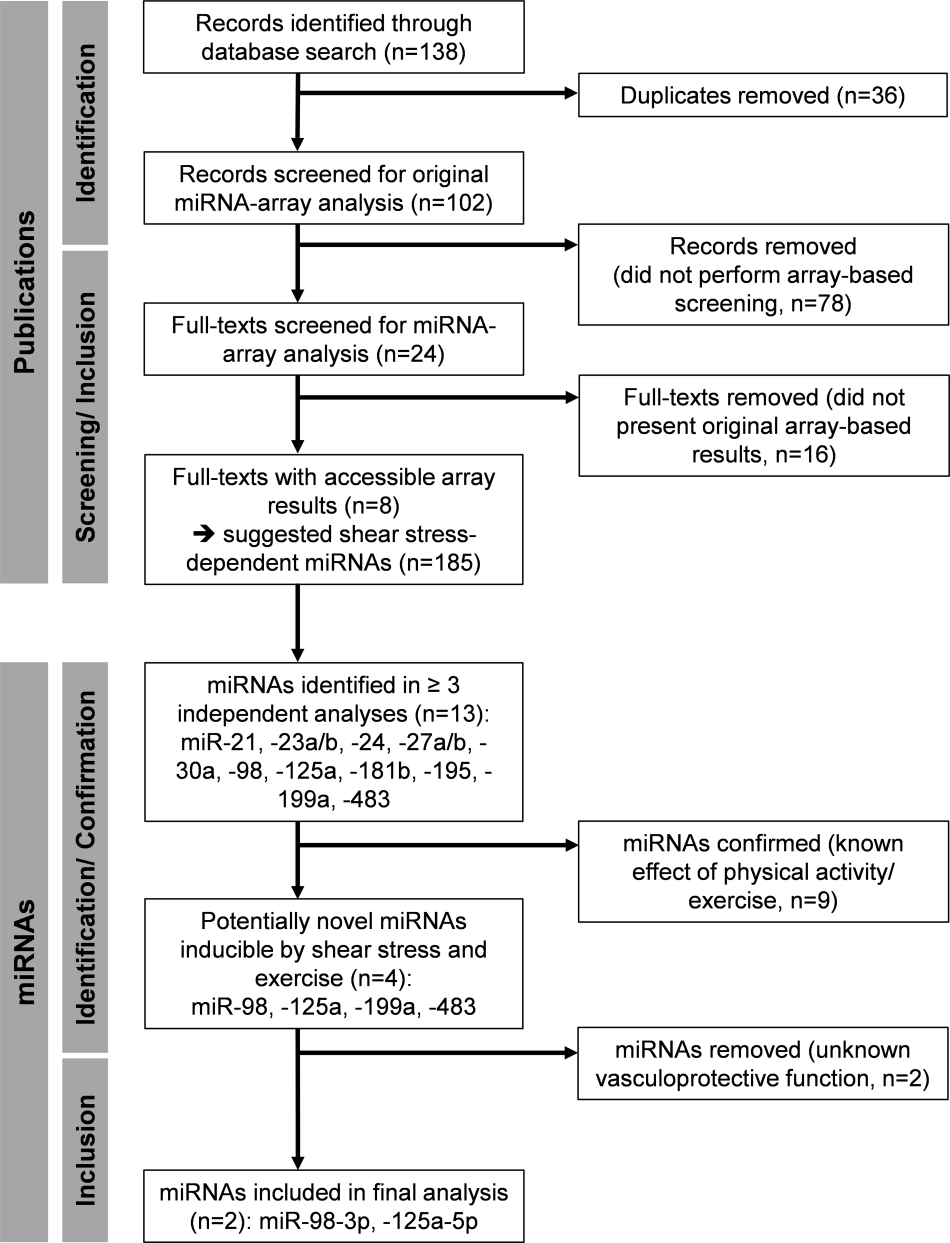
Flow chart of the array-based miRNA identification process.

The structured literature search identified eight studies on endothelial shear stress experiments presenting array-based data [44-51]. Of these, one reported data from *in vivo* shear stress experiments performed in rats [45], while the remaining seven studies reported *ex vivo* or *in vitro* data from cells of different origin [44, 46-51]. No study reported measurement of miRNAs in culture medium or blood. Combined analysis of the original data suggested 185 different shear stress-dependent miRNAs regulated in endothelial cells (see Supplementary Table 1, also for conditions and additional information). Of these, 13 miRNAs were identified in ≥ 3 independent data sets including miR-21, -23a/ b, -24, -27a/ b, -30a, -98, -125a, -181b, -195, -199a and -483 (Figure 2). Of note, application of additional selection criteria including known shear rate or duration (Supplementary Figure 1) did not improve the selection process in that no common miRNAs were identified under these combined criteria in ≥ 3 independent data sets. The second literature search on the identified miRNAs revealed that 9 miRNAs had already been associated with physical activity or exercise training in healthy humans, suggesting validity of the approach. Thus, the remaining miR-98, -125a, -199a and -483 were identified as potentially novel shear stress-dependent and exercise-regulated miRNAs (Figure 2). A final analysis on known vasculoprotective functions of the identified miRNAs revealed miR-125a-5p and miR-98-3p with anti-inflammatory and anti-atherosclerotic potential involved in the inhibition of nuclear factor-κB signaling and reduction of oxidized low-density lipoprotein (ox-LDL) uptake [44, 52, 53]. Due to missing or insufficient reports on vasculoprotective functions of miR-199a and -483, these miRNAs were not included in further analyses.

### HIIT effects on exercise miR-125a-5p and -98 levels

**Figure 3:**
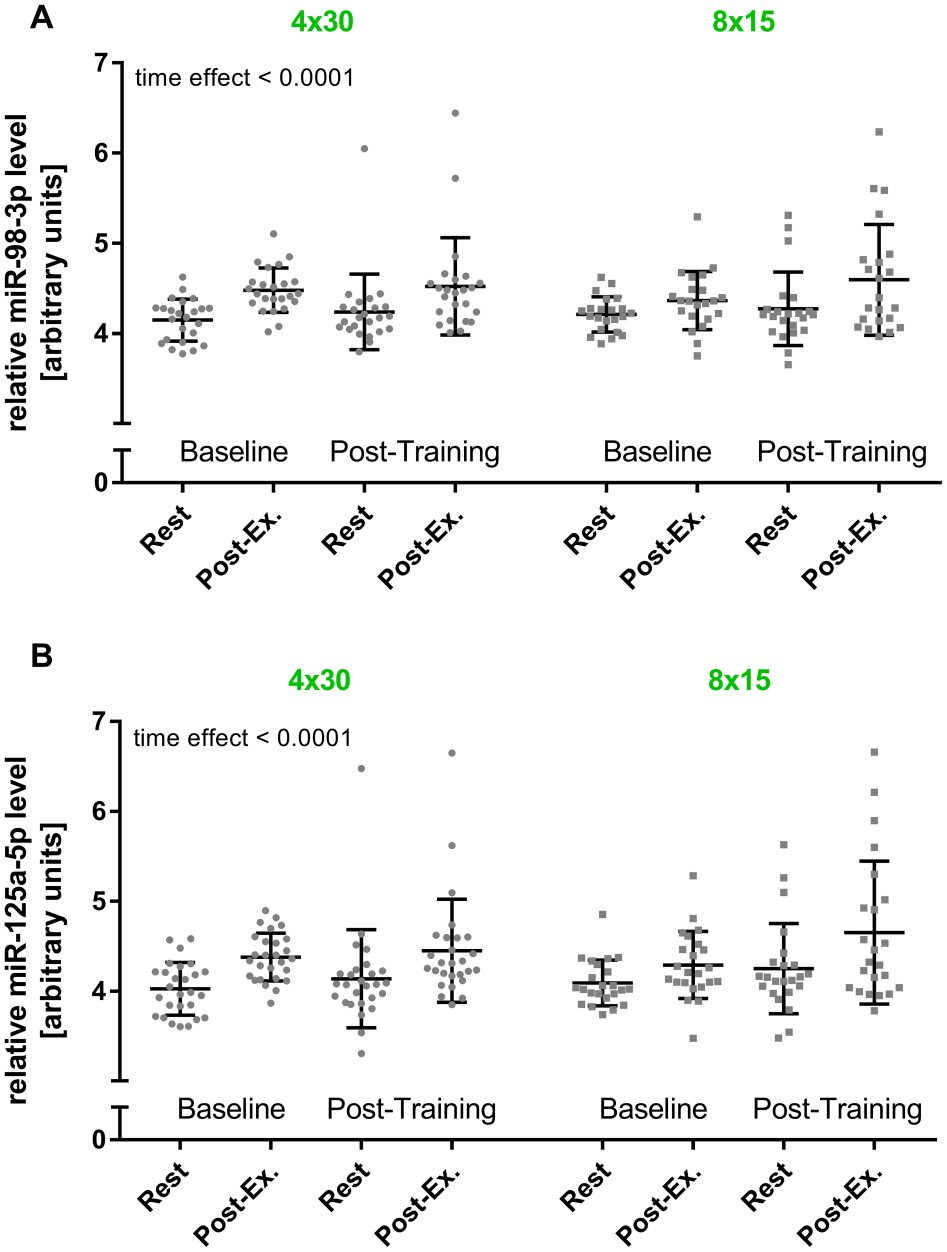
HIIT effects on miR-98-3p and miR-125a-5p levels by training group. The acute exercise effect before (baseline) and after the intervention (post-training) was determined for **(A)** miR-98-3p and **(B)** miR-125a-5p by training group. A significant increase for both miRNAs was detected (time effect), while no group or interaction effect was detected using repeated measures two-way ANOVA. Each participant is represented by one data point. Data are presented as mean ± SD.

**Figure 4:**
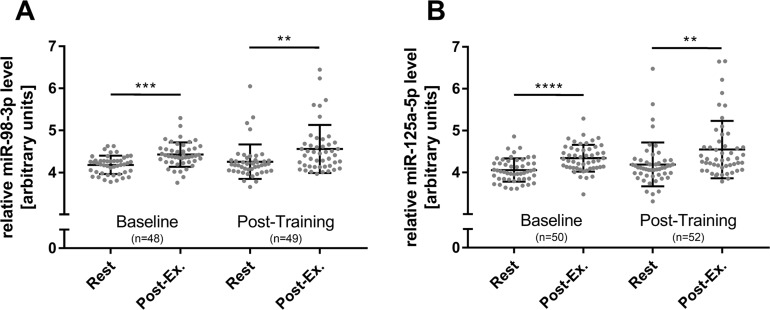
Overall HIIT effects on miR-98-3p and miR-125a-5p levels. The acute exercise effect before (baseline) and after the 4-week intervention (post-training) was determined for **(A)** miR-98-3p and **(B)** miR-125a-5p. A significant increase for both miRNAs was detected at baseline and post-training immediately after acute exercise. miRNA resting levels were not affected by the intervention. Each participant is represented by one data point. Data are presented as mean ± SD. P-values were determined using repeated measures ANOVA and post-hoc comparison. ^****^p < 0.0001; ^***^p < 0.001; ^**^p < 0.01; otherwise not significant.

In a previous study, we reported that elevated levels of c-miRNAs can already be detected after four 30 s bouts of high-intensity running at all-out speed (4 x 30 HIIT) [35]. In the current study, we assessed if HIIT with shorter work durations would also be sufficient to induce elevated c-miRNA levels. Therefore, we compared the established 4 x 30 HIIT protocol with a time and workload matched 8 x 15 s HIIT protocol. To determine changes in miRNA levels in response to HIIT, blood samples were collected at four different time points, immediately before (rest) and directly after the first (baseline) and the last training session (post-training). The separate analysis of the two HIIT groups suggested a significant effect of high-intensity running on miR-98 and miR-125a-5p levels (overall time effect, both p < 0.0001, Figure 3). However, a significant group or interaction effect was not detected. Subsequent combined analysis of both groups showed significantly elevated miR-98 and miR-125a-5p levels in response to acute exercise at baseline and at follow-up (all p < 0.01, rest vs. post-exercise, Figure 4). Comparison of acute rest vs. post-exercise miRNA levels at baseline and follow-up in terms of fold change (ΔΔCt method with resting levels as control) suggested a mean fold change of 2.02 (baseline) and 1.67 (follow-up) for miR-98 and a mean fold change of 2.6 (baseline) and 2.01 (follow-up) for miR-125a-5p. Of note, a sustained effect on resting miRNA levels was not observed.

### Shear stress effects on endothelial miRNA levels

**Figure 5:**
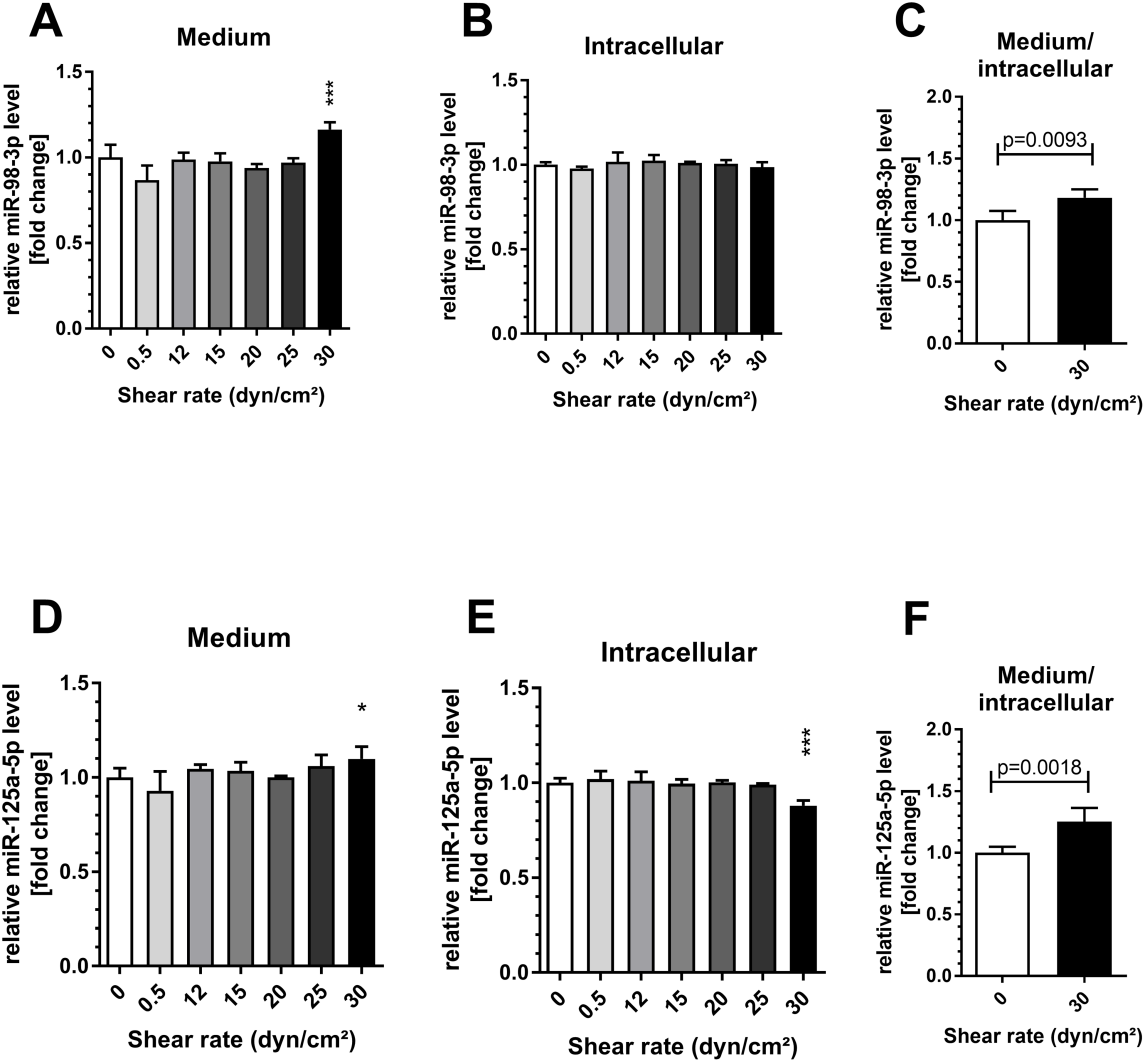
Shear stress induced endothelial miR-98-3p and miR-125a-5p secretion. Human umbilical vein endothelial cells (HUVECs) were exposed to different shear rates up to 30 dyn/cm^2^ for 1 h. A significant elevation of **(A)** miR-98-3p and **(D)** miR-125a-5p levels in the endothelial medium was detected at 30 dyn/cm^2^. Intracellular levels of **(B)** miR-98-3p remained unchanged while cellular levels of **(E)** miR-125a-5p showed a significant decrease. **(C/F)** The ratio of medium to cellular level was increased for both miRNAs. Data are presented as mean fold change ± SD. P-values are individual shear rates compared to static control (0) using ANOVA and post-hoc comparison or two-sided t-test. ^***^p < 0.001; ^*^p < 0.05.

**Figure 6:**
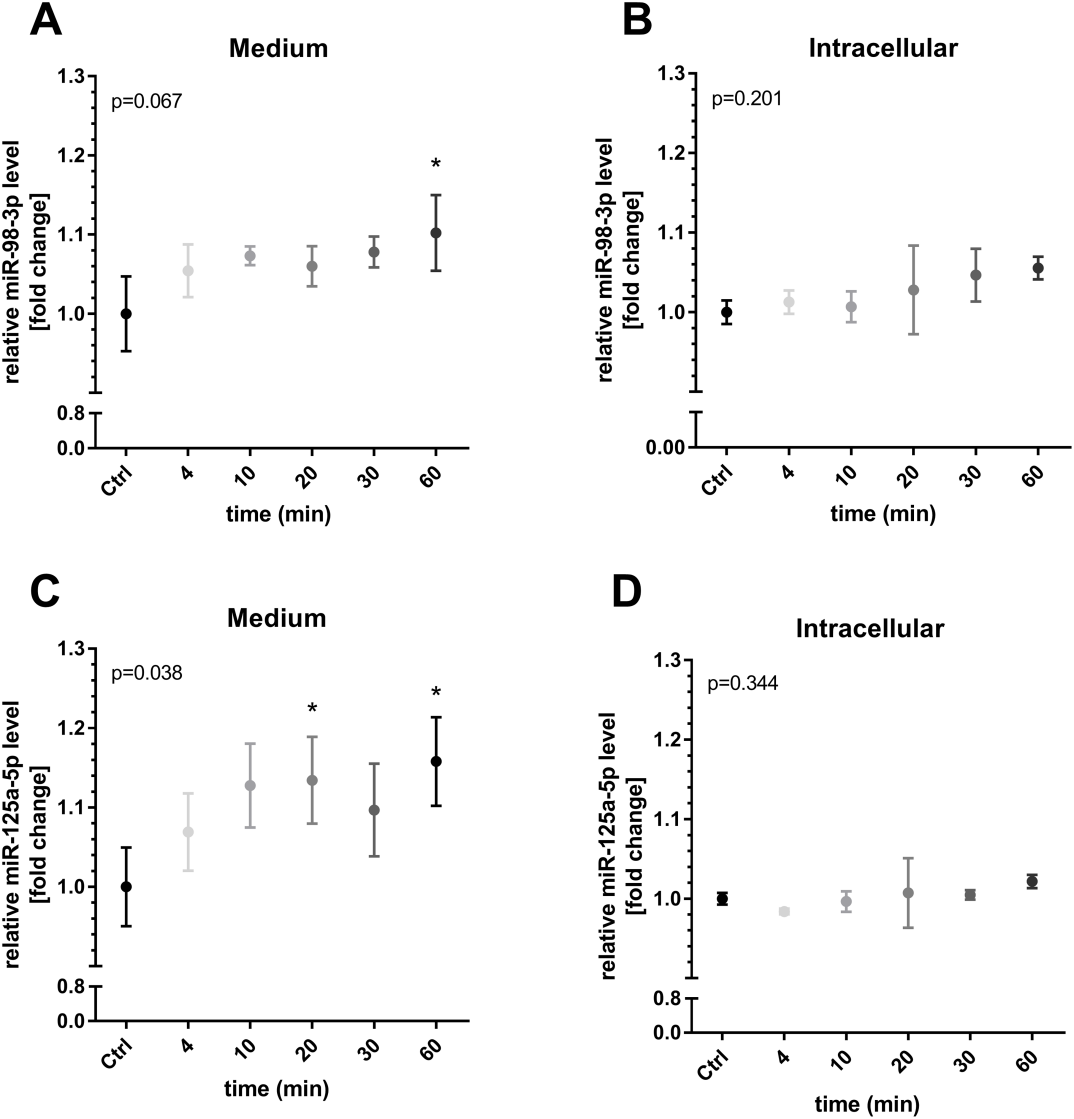
Time-dependent miR-98-3p and miR-125a-5p secretion during shear stress. HUVECs were exposed to a shear rate of 30 dyn/cm^2^ for different periods. An elevation of **(A)** miR-98-3p and **(C)** miR-125a-5p in the medium was detected with significance after 60 min. Intracellular levels of **(B)** miR-98-3p and **(D)** miR-125a-5p remained unchanged. Data are presented as mean fold change ± SD. P-values represent results of overall ANOVA. ^*^p < 0.05, post-hoc comparison to static control (Ctrl, 60 min).

The *in vitro* investigation of increasing shear rates revealed that both miRNAs, miR-98 and miR-125a-5p were significantly elevated in medium from human umbilical vein endothelial cells (HUVECs) at 30 dyn/cm^2^ (both p < 0.05 compared to control, 1 h, Figure 5). Intracellular levels of miR-98 remained unchanged while cellular levels of miR-125a-5p decreased significantly (p < 0.001, compared to control, Figure 5). Of note, lower shear rates did not affect extracellular or intracellular levels of both miRNAs. In addition, HUVECs were exposed to a shear rate of 30 dyn/cm^2^ for different periods to investigate time-dependent miRNA changes. An elevation of miR-98-3p and miR-125a-5p in the medium was detected with significance after 60 min (both p < 0.05, Figure 6). Intracellular levels of miR-98-3p and miR-125a-5p remained unchanged.

## DISCUSSION

In this study, we performed a joined analysis of array-based data in combination with *in vivo* and *in vitro* data to identify novel shear stress-dependent miRNAs potentially involved in vasculoprotective effects induced by HIIT. In brief, our main findings are 1) 13 miRNAs were differentially regulated in ≥ 3 independent array-based data sets from endothelial shear stress experiments, 2) of these, 9 had already been described in exercise training in healthy humans, 3) novel miR-98-3p and miR-125a-5p had reported vasculoprotective functions and were elevated in response to all-out high-intensity running, 4) *in vitro* experiments revealed that endothelial secretion of miR-125a-5p and -98-3p was induced by shear rates of 30 dyn/cm^2^ already after 20 and 60 min, respectively.

During the investigation of array-based data from endothelial shear stress experiments we observed considerable heterogeneity with respect to the identified miRNAs (Supplementary Table 1). Besides the different experimental conditions applied (endothelial cell type, shear rate, time), this result may also have been biased by the coverage of the used array, which has been broadly extended since the completion of the first studies [46-48, 50, 51] and the more recent studies included in our analysis [44, 45, 49]. Of note, 9 out of 13 endothelial miRNAs identified in ≥ 3 studies had already been associated with physical activity or exercise training in general, which could be interpreted as a proof of concept for our current approach. Interestingly, circulating miR-21-5p, 27a-5p [54], mir-181b-5p [55] and mir-195-5p [56] had also been identified by microarray or whole transcriptome analysis from human blood after acute exercise. With specific respect to HIIT, circulating miR-21 has been reported to be upregulated by a number of independent studies, including trained males performing 4 × 30 s all-out cycling bouts which showed an acute elevation in miR-21 plasma levels [57] and moderately trained males performing 10 x 60 s cycling intervals at peak power output, which showed an acute elevation of miR-21 in exosomes [58]. Out of the remaining 4 miRNAs, miR-98, -125a-5p, -199a and -483 had not been reported to be regulated in response to exercise in healthy humans. Of note, one recent study reported down regulation of miR-125 family member miR-125b-5p during sprint training (18 all-out 15 m sprints, 17 s of passive recovery) [59]. However, the group did not determine miR-125a-5p levels and future studies are needed to investigate potential differences of miR-125 family members in response to exercise.

After miRNA identification and selection in step 1 and evaluation of reported vasculoprotective capacity, we selected miR-98-3p and miR-125a-5p (for details see below) for subsequent *in vivo* and *in vitro* analysis. miR-199a and miR-483 were not analyzed further in this study as reports on vasculoprotective capacity were missing or insufficient. Circulating miR-98-3p and -125a-5p levels both showed an acute increase in response to a single 4-min HIIT session already at baseline. The effect was also detected after the last training session at follow-up, which might support the conclusion that miR-98-3p and -125a-5p levels were acutely induced during the entire training period even though not all training sessions were monitored. Moreover, resting levels of both miRNAs remained unchanged, suggesting direct stimulation by acute exercise and associated mechanisms without sustained effects. Of note, and in contrast to our initial assumption, the observed increased levels were independent of the training group. Thus, we can conclude that a single session of 2 min all-out running HIIT is sufficient to induce miR-98-3p and -125a-5p, while it remains elusive if longer work/rest intervals produce different levels of miR-98-3p and -125a-5p. The observed rapid acute increase of c-miRNA levels, which is also in line with our previous investigations [35, 36] points to a release or secretion mechanism as *de novo* expression would most likely require more time [61]. Subsequently, we tested both miRNAs in an *in vitro* endothelial shear stress model using periods from 4 to 60 min and different shear rates. We found significantly elevated levels of miR-98-3p and -125a-5p in the conditioned medium of HUVECS cultured at 30 dyn/cm^2^. A time series with cells kept at 30 dyn/cm^2^ revealed that miR-125a-5p was already upregulated after 20 min while both miRNAs were elevated in the media after 1 h. Of note, the initially identified array-based studies did not report measurement of miRNAs in cell culture media and, to the best of our knowledge, we are first to determine secretion of miRNAs including miR-98-3p and -125a-5p in *in vitro* shear stress experiments with periods of 4 to 60 min. This might be of central interest as previous studies using endothelial shear stress models did not report experimental durations similar to acute bouts of exercise and the generated data was thus not well-translatable to *in vivo* conditions. Our results provide further evidence that shear stress during exercise likely causes endothelial secretion of miRNAs *in vivo* and that the time point of miRNA determination is an important aspect in the measurement of circulating miRNAs. Interestingly, we also did not detect an increase in cellular miRNAs levels during the experimental period and future *in vitro* shear stress studies should also investigate the amounts of secreted miRNAs to gain better insight into the involved (time-dependent) secretion mechanism and cellular reuptake in the applied model.

It is also of interest to compare our *in vitro* findings with investigations of hemodynamic forces induced by exercise *in vivo*. It has been suggested that 30 dyn/cm^2^ may already be reached in large arteries at submaximal exercise intensities [63]. As our *in vitro* study primarily aimed at investigating if endothelial cells secrete miR-98-3p and -125a-5p into the medium and at determination of the earliest time point at which elevated c-miR levels can be detected, we do not know if higher shear rates, most likely induced by high-intensity running, would have induced even higher c-miR levels. While our *in vitro* experiments reveal that both miRNAs are shear stress-responsive it seems conceivable that also submaximal exercise intensities could result in a significant elevation of miR-98-3p and -125a-5p levels. However, *in vivo* miR-98-3p and -125a-5p elevation may also depend on additional effectors and may also be influenced by the intermittent nature of HIIT. Further research is therefore needed to investigate which additional stimuli affect miRNA levels *in vivo* and to determine selected miRNA levels after each individual HIIT bout for a better determination of work/ rest duration effects.

With regard to vasculoprotective functions, it has been reported that miR-98-3p exerts anti-inflammatory effects in endothelial cells by inhibition of nuclear factor–κB signaling [44] and that miR-125a-5p may reduce inflammatory cytokines in monocytes [52]. Accordingly, both miRNAs have been reported to reduce ox-LDL uptake in endothelial cells and monocytes, respectively [52, 53], and may thus prevent atheroma development and reduce the overall atherosclerotic burden. In addition, Verjans et al. [64] recently identified miR-125a-5p in an extensive screening of heart failure-associated miRNAs as an inducer of non-pathological hypertrophic growth, while protecting the adult pressure-overloaded heart through increased cardiac contractility. Future studies will be needed to investigate if physical exercise also induces miR-125a-5p uptake or production in the heart. With regard to miR-199a which had not been included in our analysis, we became aware of a report by Joris et al. [60] during preparation of this manuscript, which provided functional data suggesting that miR-199a-3p and -5p take part in the regulation of the endothelial NO synthase. It may thus modulate key endothelial functions such as angiogenesis and vascular tone, suggesting miR-199a as interesting candidates for future investigations. Of note, a potential cancer-related function of miR-98-3p and miR-125a-3p have most recently been reported. Russo et al. [65] suggested an oncosuppressive role for microRNA-125a based on its antiproliferative activity and overexpression of mir-125a may predict better survival in cancer patients [66]. In addition, miR-98 has been involved in the response to treatment of patients with advanced colorectal cancer. Thus, future studies should focus on elevated miR-98 and miR-125a levels in different training modalities and their potential mediation of a preventive effects on both, cardiovascular diseases and the development of different cancer types.

## MATERIALS AND METHODS

### Study design

The current investigation was based on a three-step study design (Figure 1). In step one, a comprehensive literature search was conducted to identify miRNA array data from endothelial shear stress experiments. Meta data was then searched for shear stress-sensitive miRNAs reported in ≥ 3 independent studies. In step two, identified candidate miRNAs were analyzed in two different HIIT groups of healthy individuals immediately after acute exercise and pre- post-intervention. In step three, identified miRNAs were analyzed at different shear rates and time points using an *in vitro* endothelial shear stress model for validation.

### Literature search and identification of miRNAs

#### Search strategy and eligibility criteria

To identify publications providing results of array-based miRNA screenings of endothelial shear stress experiments, a structured literature search was conducted using PubMed (MEDLINE database) for records published until July 2018. Combinations of the following key words were used: “microRNA”, “miRNA”, “shear”, “stress” and variations thereof. Manual searches were also performed based on references from identified articles. The individual steps of report identification, screening and processing are documented in the study flow-chart (Figure 2). Any article reporting results of an array-based screening of endothelial shear stress-induced miRNAs was considered for the analysis. Articles had to be original research (not a review, book [chapter], conference abstract, thesis or website) and be written in English. Articles were excluded if processed array results (i.e. a list of significantly regulated miRNAs) was not available. Results were extracted including information on type of endothelial cells used, applied shear rate (dyn/cm^2^), time under shear stress and suggested miRNAs (by identification number) including strand information (-3p, -5p) if available. The originally applied p-values and thresholds (magnitude of change) to identify regulated miRNAs were adopted as reported in the original investigation. Screening and extraction of data was performed by two reviewers (BS and FB).

#### Identification of shear-stress induced miRNAs

The extracted data was analyzed for endothelial miRNAs suggestively regulated in ≥ 3 independent studies. Positive identification at this step was also accepted if strand type was different (i. e. -3p, -5p) or not specified (Supplementary Table 1). This was done since a number of identified studies did not specify miRNA strand identity. In a second literature search, reports on the identified miRNAs in combination with physical exercise in healthy humans were investigated (Figure 2). The search was conducted using PubMed for records published until August 2018. Combinations of the following key words were used: “miR-[identification number of the respective miRNA]”, “miRNA”, “physical activity”, “exercise”, “sport” and variations thereof. Manual searches were also performed based on references from identified articles. In a final selection process, identified miRNAs were analyzed towards reported vasculoprotective functions (Figure 2). At this step of the analysis, miRNA strand type information was included for selection (-3p was considered if not indicated). All available records of the respective miRNAs were screened and full-texts of records indicating functional analysis linked to vasculoprotective activity of the respective miRNA were assessed. Reports were evaluated by two reviewers (BS and FB) and the final selection of miRNAs was discussed based on the identified functional evidence until consensus was reached.

### Participants and exercise testing

#### Inclusion criteria and randomization

A randomized controlled interventional study was used to analyze the effects of two different 4-week HIIT programs in young healthy moderately trained individuals on circulating levels of the selected miRNAs. Samples were drawn at rest and post-exercise immediately after baseline and follow-up high-intensity running. The detailed study protocol and changes in exercise performance parameters in response to the intervention have been reported elsewhere [36]. In brief, 66 female and male students > 18 years were recruited at the Institute of Sports Medicine of the University Hospital Muenster. All investigations were performed in accordance with the declaration of Helsinki and after the approval of the local ethics committee of the medical association Westfalen-Lippe and the Westphalian Wilhelms-University of Muenster (project-no. 2013-231-f-S, study acronym SPORTIVA). Written informed consent of participants was obtained prior to subjects’ participation in the study. In total, 14 participants dropped out of the study, 7 from the 4 x 30 HIIT group and 7 from the 8 x 15 HIIT group due to injury/ illness not associated with the intervention and scheduling problems at retest. Thus, 27 participants of the 4 x30 HIIT group and 25 participants of the 8 x 15 HIIT group were included in the current analysis (Figure 2). Characteristics of the analyzed participants were as follows: female = 71.7 %, male = 28.3 %, age = 22.05 ± 1.78 years, height = 174.21 ± 7.29 cm, body mass = 67.39 ± 10.65 kg, maximal speed in ICRT = 15.51 ± 1.87 km∙h^-1^, speed at individual anaerobic [lactate] threshold (IAT) = 11.17 ± 1.07 km∙h^-1^, maximal blood lactate concentration = 12.69 ± 2.65 mmol∙L^-1^, maximal heart rate 196.98 ± 8.17 bpm, and as reported elsewhere [36].

#### Exercise test procedures

Exercise parameters at baseline and follow-up were determined using the ICRT field test protocol (maximal performance test) as described [67, 68] with modifications [62]. The test started at 8.0 km·h^-1^, increasing by 2.0 km·h^-1^ every 3 min until total exhaustion of the participant. The pace was indicated by an automated acoustic device. Blood was sampled from participants’ earlobes for blood lactate concentration measurement (20 μl heparinized capillary; Biosen S-line, EKF Diagnostics, Magdeburg, Germany) after each interval (3 min). Performance at IAT (baseline lactate + 1.5 mmol∙L^-1^) was calculated using Winlactat software version 5.0.0.54 (Mesics, Muenster, Germany) as described [36]. Participants were then allocated to one of two training groups by stratified block randomization using sex and performance (i. e. power output [running speed] at IAT) as primary parameters [36]. The ICRT and all HIIT sessions were performed indoors on a synthetic 200 m running track at ambient temperature (18 – 22°C, ~ 60 m above sea level).

#### HIIT interventions

During the 4-week training intervention participants performed two controlled exercise sessions per week (one day off between sessions) supervised by at least one experienced trainer. All training sessions started with a warm-up phase of 10 min including running at ~8.0 m·h^-1^ and light stretching. A cool-down phase was not included. Both HIIT protocols were matched for time and total workload.

4 x 30 HIIT: participants were instructed to run at maximal speed for 4 x 30 s (all-out) with 30 s of active recovery periods at warm-up speed between bouts.

8 x 15 HIIT: participants were instructed to run at maximal speed for 8 x 15 s (all-out) with 15 s of active recovery periods at warm-up speed between bouts.

Additional blood sampling for miRNA quantification was performed at the first and the last HIIT sessions at rest and immediately post-exercise.

### Cell culture and shear stress experiments

Human umbilical vein endothelial cells (HUVECs) from 3 donors were collected as reported previously [69, 70]. Cells were grown to confluence at 37°C with 5% CO_2_ on cross-linked gelatin-coated culture plates in endothelial cell growth medium (ECGM; Promocell, Heidelberg, Germany) containing the supplement mix C-39-210 (Promocell) supplemented with 50 mg/ml streptomycinsulphate and 50 U/ml penicillin G (Invitrogen, Darmstadt, Germany). Cells were used for shear stress experiments performed in the cone-and-plate “BioTech Flow” (BTF)-System at 37°C, 5% CO_2_ as described [71]. The BTF-System provides constant laminar homogenous flow through circulation of medium provoked by a cone above the culture plate [72]. The inner 10-mm radius of the culture plate was kept free of cells due to non-defined shear rate in the center of the plate. Medium viscosity was increased using 3% polyvinylpyrrolidone (MW 360,000; Sigma-Aldrich, Munich, Germany) [71]. Subsequently, cells were exposed to different shear rates for up to 1 h in n = 3 independent culture plates simultaneously. Shear rates ranged from 0.5 to 30 dyn/cm^2^. For *in vitro* time series analyzes (4 to 60 min, n = 3 for each time point), HUVECs from 3 different donors were treated as stated above and were exposed to 30 dyn/cm^2^.

### miRNA extraction and quantification

Blood sampling from participants’ earlobes was performed as reported previously [35, 36, 73] immediately at the testing site using a 20 μl K2 EDTA capillary (Sarstedt, Nuernbrecht, Germany) and RNA was extracted using 750 μl peqGOLD TriFast (VWR, Darmstadt, Germany) according to the manufacturer’s instruction. The applied method allows the detection of acute changes in c-miRNA levels immediately after exercise and prevents the bias of hemolysis. Each sample was immediately supplemented with 10 nM *Caenorhabditis elegans* cel-miR-39-3p spike-in control following manufacturer’s instruction (Thermo Fisher Scientific, Darmstadt, Germany) for normalization as reported [35, 74, 75]. RNase-free glycogen (70 μg/sample; VWR) was used as carrier to optimize extraction efficiency [76]. Isolated RNA was resuspended in 20 μl of nuclease-free water. RNA from cultured HUVECs was extracted using 2.0 ml peqGOLD TriFast and processed according to the manufacturer’s instruction. From each experimental condition, the culture medium was collected completely and RNA from 20 μl conditioned medium was extracted using 750 μl peqGOLD TriFast as described above and resuspended in 20 μl RNase-free water. Quantification of mature hsa-miR-98-3p, hsa-miR-125a-5p, hsa-miR-222-3p, and cel-miR-39-3p was performed by quantitative real-time polymerase chain reaction (qRT-PCR) using 5' adaptor ligation and target-independent cDNA generation in a single reaction (TaqMan Advanced MicroRNA technology; Thermo Fisher Scientific, Darmstadt, Germany). In brief, 1.0 μl of RNA solution was used for adaptor ligation and reverse transcription according to manufacturer’s instructions. cDNA was diluted 1:10 in ultra-pure water und 1.25 μl were used for final qRT-PCR reactions performed in a 384-well format in duplicates on an ABI7500 fast RT-PCR system (Life Technologies, Carlsbad, USA). Relative quantification was performed using the ΔCt method and miR-98-3p and miR-125a-5p values were expressed as (1/ΔCt)*100 for presentation. For cell culture experiments, cel-miR-39-3p spike-in control was used for analysis of culture medium and hsa-miR-222-3p was used as endogenous reference for analysis of cellular miRNA levels. Duplicates with a difference greater than 2 Ct were excluded from the analysis. The number of included samples is presented in Figure 4.

### Limitations

Some limitations for the current investigation might exist. First, our findings may not be directly translated to other groups or populations as our results for HIIT were detected in a population of young healthy female and male Caucasians. Second, the presented results are based on the determination of circulating miRNAs from blood. As miR-125a-5p and miR-98-3p might be concentrated in micro vesicles or associated to carrier proteins with the potential to shuttle into target cells with high efficiency, future studies are needed to investigate the effect of physical exercise on miRNA elevation in the blood. Moreover, additional studies are warranted to analyze potential additional regulators of miR-125a-5p and miR-98-3p such as hypoxia. We have applied a spike-in control (cel-miR-39) for our *in vitro* and *in vivo* analysis and it might be necessary to validate our findings in future studies once an appropriate endogenous (exercise-independent) miRNA has been identified.

### Statistical data analysis

For the initial identification of miRNAs from available array-based studies, extraction spread sheets were generated using Microsoft Excel (Microsoft, Redmond, USA) to identify miRNAs reported by ≥ 3 studies. *In vivo* and *in vitro* statistical data analyses were performed using GraphPad PRISM V7.0 software (GraphPad Software Inc., La Jolla, USA). Data are presented as mean ± SD. Data were tested for normal distribution using D’Agostino-Pearson normality test (omnibus K2 test). Training effects on miRNA levels were determined using repeated measures two-way ANOVA (time x HIIT group). In addition, overall HIIT effects (both groups) were analyzed using repeated measures ANOVA (4 time points). *In vitro* shear stress experiments were analyzed using ANOVA or two-sided t-test when indicated and presented as fold change compared to respective control. Bonferroni's multiple comparisons test was used. Friedman test and Dunn's multiple comparisons test were used when indicated. Significance was declared at p < 0.05

## CONCLUSIONS

We conclude that re-analysis of miRNA array results such as data from endothelial shear stress experiments can be used to identify miRNAs inducible by exercise. Our data provides evidence that the identified miR-98-3p and miR-125a-5p can be rapidly secreted by endothelial cells *in vitro* and that endothelial cells might be the source for increased circulating miR-98-3p and miR-125a-5p levels in response to HIIT *in vivo*. Since both miRNAs attenuate endothelial inflammation, miR-98-3p and miR-125a-5p may mediate vasculoprotective effects of physical exercise including HIIT.

## SUPPLEMENTARY MATERIALS FIGURE AND TABLE





## Abbreviations

BTF: BioTech Flow (System)
c-miRNA: circulatory microRNA
HIIT: high-intensity interval training
HUVEC: human umbilical vein endothelial cell
IAT: individual anaerobic threshold
ICRT: incremental continuous running test
miRNA: microRNA
NO: nitric oxide
ox-LDL: oxidized low-density lipoprotein
qRT-PCR: quantitative real-time polymerase chain reaction
