# Supplementary material for: A three-step approach identifies novel shear stress-sensitive endothelial microRNAs involved in vasculoprotective effects of high-intensity interval training (HIIT)
Source: Oncotarget. 2019 Jun 4;10(38):3625–40. (PMC6557206)
Supplement: Supplementary file 1 [file oncotarget-10-3625-s001.pdf]

A three-step approach identifies novel shear stress-sensitive endothelial microRNAs involved in vasculoprotective effects of high-intensity interval training (HIIT)

SUPPMENTARY MATERIALS FIGURE

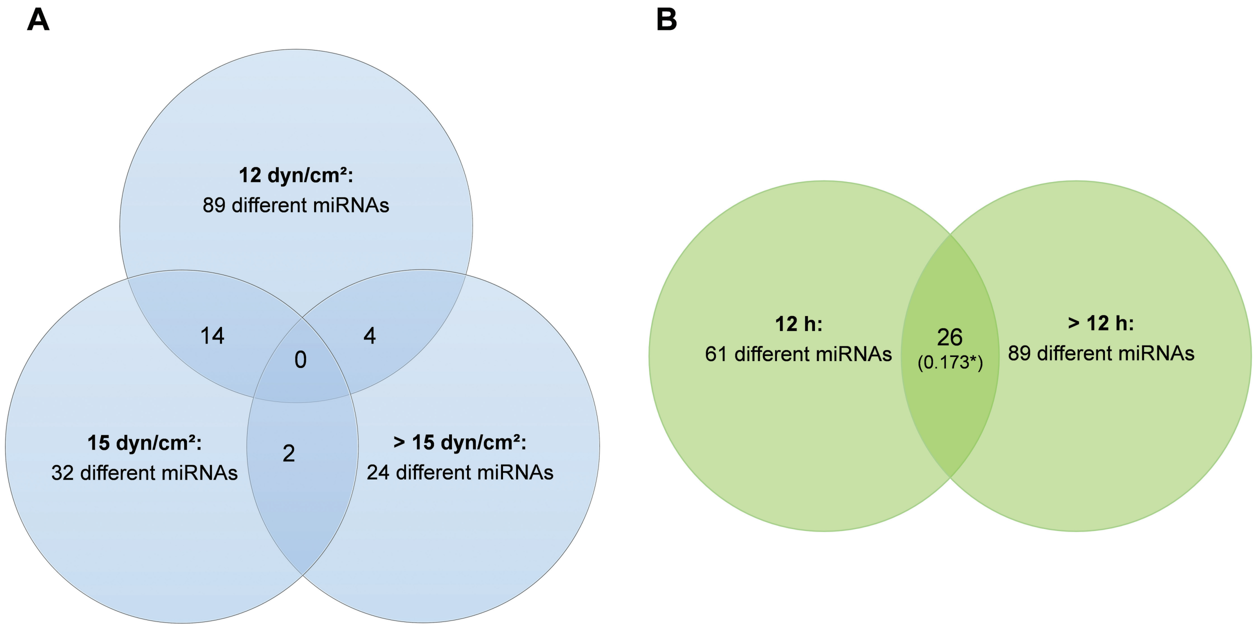

Supplementary Figure 1: Venn diagrams representing miRNA identification using array-based data. Identified endothelial shear stress-induced miRNAs from independent studies by **(A)** applied shear rate and **(B)** experiment duration (if available). No common miRNAs were identified over all three different shear rates and 26 common miRNAs for duration ≥12 h. A combination of both selection criteria did not reveal common miRNAs. * indicates Jaccard coefficient.

Supplementary Table 1:

See Supplementary File 1
